# The pineapple MADS-box gene family and the evolution of early monocot flower

**DOI:** 10.1038/s41598-020-79163-8

**Published:** 2021-01-13

**Authors:** Juan Hu, Xiaojun Chang, Ying Zhang, Xianxian Yu, Yuan Qin, Yun Sun, Liangsheng Zhang

**Affiliations:** 1 Fujian Provincial Key Laboratory of Haixia Applied Plant Systems Biology, College of Horticulture, Fujian Agriculture and Forestry University, Fuzhou, 350002 China; 2 School of Urban-Rural Planning and Landscape Architecture, Xuchang University, Xuchang, 461000 China; 3 Genomics and Genetic Engineering Laboratory of Ornamental Plants, College of Agriculture and Biotechnology, Zhejiang University, Hangzhou, 310058 China

**Keywords:** Plant development, Plant evolution, Plant sciences

## Abstract

Unlike the flower of the model monocot rice, which has diverged greatly from the ancestral monocot flower, the pineapple (*Ananas comosus*) flower is more typical of monocot flowers. Here, we identified 43 pineapple genes containing MADS-box domains, including 11 type I and 32 type II genes. RNA-seq expression data generated from five pineapple floral organs (sepals, petals, stamens, pistils, and ovules) and quantitative real-time PCR revealed tissue-specific expression patterns for some genes. We found that *AcAGL6* and *AcFUL1* were mainly expressed in sepals and petals, suggesting their involvement in the regulation of these floral organs. A pineapple ‘ABCDE’ model was proposed based on the phylogenetic analysis and expression patterns of MADS-box genes. Unlike rice and orchid with frequent species-specific gene duplication and subsequent expression divergence, the composition and expression of the ABCDE genes were conserved in pineapple. We also found that *AcSEP1/3*, *AcAG*, *AcAGL11a/b/c*, and *AcFUL1* were highly expressed at different stages of fruit development and have similar expression profiles, implicating these genes’ role in fruit development and ripening processes. We propose that the pineapple flower can be used as a model for studying the ancestral form of monocot flowers to investigate their development and evolutionary history.

## Introduction

Typical flowers are composed of sepals, petals, stamens, and pistils arranged in concentric rings around a central axis, and the molecular and evolutionary mechanisms of floral development have been studied extensively. The genetic ‘ABC’ model of floral organ development was originally based on the functional analysis of genes in *Arabidopsis thaliana* and *Antirrhinum majus* then subsequently extended to many other flowering plants^1,2^. In *Arabidopsis*, A function is performed by *APETALA1* (*AP1*) and *APETALA2* (*AP2*), B function by *APETALA3* (*AP3*) and *PISTILLATA* (*PI*), C function by *AGAMOUS* (*AG*), D function by *SEEDSTICK* (*STK*, also called *AGAMOUS-LIKE11* [*AGL11*]) and *SHATTERPROOF*(*SHP*), and E function by *SEPALLATA1/2/3/4* (*SEP1/2/3/4*, also known as *AGAMOUS-LIKE2/4/9/3* [*AGL2/4/9/3*]). According to the ABC model, floral organs in each of the four whorls are specified by three classes of genes with different homeotic functions acting in a combinatorial way. Specifically, A-function genes alone specify sepals; A and B petals; B and C stamens; and C alone carpels. Moreover, A and C genes function antagonistically^1,3,4^. The classic ABC model was modified by adding D and E functions, leading to the ABCDE model^5^. D-function genes specify ovule identity, and E-function genes contribute to the development of all four whorls by interacting with A-, B-, C-, and D-class genes in the form of heterotetramer protein complexes^5^.

Monocot flower morphology and the underlying developmental mechanisms have notable similarities and differences with those of early-diverging angiosperms and eudicots. The four predicted A-class genes (AP1/FUL-like subfamily) in rice (*Oryza sativa*) are *OsMADS14*, *OsMADS15*, *OsMADS18*, and *OsMADS20*^6,7^. *osmads14 osmads15* double mutants exhibit true recessive phenotypes with defects in the first and second whorls, consistent with the phenotypes of A-function mutants in *Arabidopsis*^7^. *OsMADS2*, *OsMADS4*, and *OsMADS16/ SPW1* are B-function genes and primarily determine the identity of lodicules and stamens in rice^8^. *OsMADS16/SPW1* is homologous to the *AP3* gene of *A. thaliana* and has a similar expression domain and analogous function. The *PI* homologs *OsMADS2* and *OsMADS4* diverge in terms of B function^8,9^. *OsMADS2* is specialized for the development of lodicules and does not affect stamen morphogenesis. In contrast, *OsMADS4* maintains the ancestral gene function of the B-class *PI* lineage, and suppression of its expression leads to ectopic development of palea/lemma-like organs and carpels in the second and third whorls^10,11^. *OsMADS3* and *OsMADS58* are homologous to the *Arabidopsis AG* gene, and these two copies show partial sub- and neo-functionalization^8,12^. *OsMADS3* is primarily involved in stamen and ovule development, and a deletion mutant was found to have normal carpel development^13^. A knockdown mutation in the *OsMADS58* gene led to disrupted floral meristem determinacy rather than floral organ defects. In addition to floral determinacy defects, the silencing of both *OsMADS3* and *OsMADS58* resulted in homeotic conversion of female and male reproductive organs into palea/lemma-like organs and lodicules, respectively, similar to the phenotypes of the *ag* mutant in *Arabidopsis*^13^. Two D-function genes, *OsMADS13* and *OsMADS21*, underwent functional diversification. While *OsMADS13* is primarily involved in ovule formation, *OsMADS21* lost its role in this process^14,15^. The rice genome contains five SEP-like subfamily homologs: *OsMADS1*, *OsMADS5*, *OsMADS7*, *OsMADS8*, and *OsMADS34*. Among them, *OsMADS1* and *OsMADS34* contribute to the specification of the four whorls of floral organs and control the determination of spikelet meristems^16^.

The expression and function of the ABCDE MADS-box genes are known to have helped establish the unique floral architecture of rice and orchid^17,18^. In contrast, the genetic basis of pineapple flower development and the evolutionary history of the molecular mechanisms in ancestral monocot flowers remain poorly understood. Therefore, a comprehensive study of this gene family, especially the phylogeny and roles of MADS-box genes in flower development, is urgently needed for pineapple^19^. To classify the MADS-box genes in pineapple and elucidate their evolutionary relationships, we utilized the pineapple genome as a reference for a systematic phylogenetic analysis. We also studied the expression patterns of pineapple MADS-box genes at different stages of flower and fruit development. We identified candidate floral ABCDE genes in pineapple, and the gene numbers and expression patterns could explain the conserved floral architecture of pineapple flower to a large extend.

## Results

### Pineapple flower and the ancestral monocot flower

**Figure 1:**
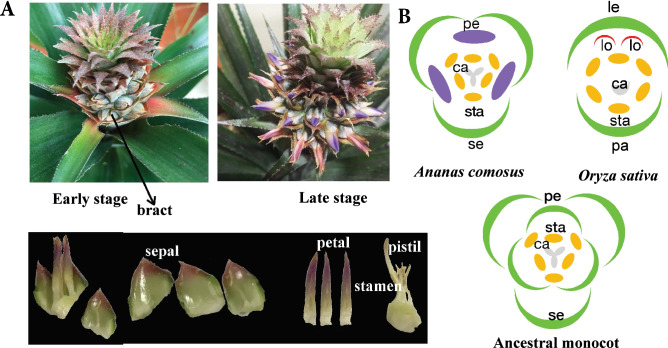
Floral organs of pineapple and floral diagrams of pineapple, rice, and the ancestral monocot. (**A**) Inflorescence of pineapple. Dense pineapple flowers are arranged in a spiral at the periphery of the spadix rachis. Each flower comprises sepals, petals, stamens, and pistils and is protected by one thick bract. (**B**) Floral diagrams of pineapple, rice, and the proposed ancestral monocot. Individual pineapple flowers are trimerous with three sepals (green) and petals (purple) in the two outer whorls, six stamens arranged in two whorls with three organs in each, and one pistil with three fused carpels in the center. In rice, there are two outer perianth organs (green, adaxial palea and abaxial lemma); two lodicules (red) internal to the lemma, corresponding to petals in non-grasses; six stamens in one whorl; and a single carpel. The floral structure of the ancestral monocot is similar to that of pineapple, except for the two outer whorls of perianth organs. *se* sepal, *pe* petal, *sta* stamen, *pa* palea, *le* lemma, *lo* lodicule, *ca* carpel.

Pineapple, orchid, and rice vary vastly in terms of flower morphology. Pineapple, a perennial monocot of the Bromeliaceae family, is indigenous to Central and South America^20^. Each pineapple flower, from the outside inwards, is composed of three broadly ovate and fleshy sepals in whorl 1, three long elliptic petals in whorl 2, six stamens in whorl 3, and a pistil in whorl 4 with three fused carpels (Fig. 1A). Orchids have unique labellum and gynostemium. The grass species, including rice, have the unique floral organization and morphology of florets, which comprise grass-specific peripheral organs, including a pair of bract-like organs (lemma and palea), two lodicules, and conserved sexual organs (six stamens and a pistil with a single carpel) (Fig. 1B). Conversely, individual pineapple flowers resemble ancestral monocot flowers in their organization and arrangement, except that the ancestral flower has two outer whorls and is considered to have undifferentiated perianth organs rather than sepals and petals^21–23^ (Fig. 1B). Thus, unlike rice and orchids, which has diverged significantly from the ancestral flower, the pineapple flower is typical of monocot flowers and more closely resembles an ancestral state.

### The identification of pineapple MADS-box genes

**Figure 2:**
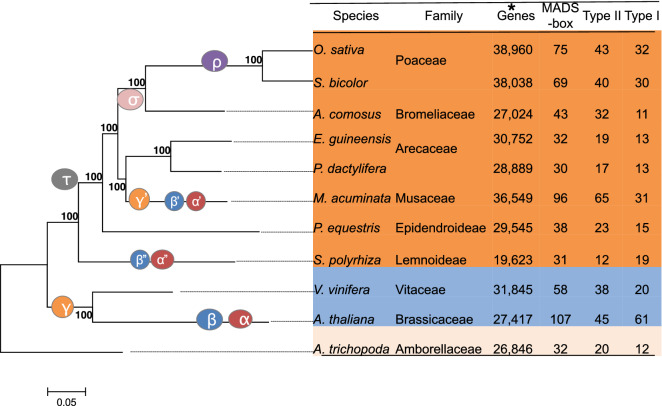
The phylogenetic tree and MADS-box gene numbers of pineapple and ten other species in the context of angiosperms. The ML phylogenetic tree of 11 species was constructed using 756 single-copy genes by orthfinder^24^. *The number of all coding genes in each species. Circles with different Greek letters represent known WGDs identified in previous studies^25–27^. The MADS-box gene number in each species and the classifications were based on the phylogenetic tree (Supplementary Figure S2).

To identify the MADS-box genes in pineapple, we employed the following strategy: a profile hidden Markov model (HMMER) search against the pineapple genome protein database using the SRF-TF domain (PF00319) as a query dialog. Redundant and extremely short sequences were removed, and conserved MADS domains were detected by verifying sequences in the Pfam databases. Forty-four candidate pineapple MADS-box proteins with a complete M-domain were obtained and designated AcMADS1 to AcMADS43 (Tables 1, Supplementary Table S1). To clarify the evolutionary relationships of MADS-box genes in various plant species, MADS-box genes from ten other plant species were detected using the same method described above for pineapple (Fig. 2). We found that the 43-member MADS-box gene family of pineapple was relatively small compared to those of other species. The pineapple MADS-box genes were subsequently designated as type I (11) and type II (32) MADS-box genes, according to the phylogenetic relationship with well-known MADS-box gene members in *Arabidopsis.*

**Table 1:** List of the ABCDE genes in the pineapple and rice genomes.

Subfamily	Pineapple				Rice	
	Gene name	Gene ID	Chromosome location		Gene name	Gene ID
AP1/FUL	*AcFUL1* *AcFUL2*	Aco004839.1 Aco012428.1	LG07 LG01	217862–228880 2127109–2132551	*OsMADS14*	LOC_Os03g54160.1
					*OsMADS15*	LOC_Os07g01820.1
					*OsMADS18*	LOC_Os07g41370.1
					*OsMADS20*	LOC_Os12g31748.1
AP3	*AcAP3*	Aco017589.1	LG09	328289–335839	*OsMADS16*	LOC_Os06g49840.1
PI	*AcPI*	Aco019365.1	LG05	6423650–6427189	*OsMADS2*	LOC_Os01g66030.1
Bsister (Bs)	*AcBS*	Aco008359.1	LG19	9790787–9794944	*OsMADS29*	LOC_Os02g07430.1
					*OsMADS30*	LOC_Os06g45650.1
					*OsMADS31*	LOC_Os04g52410.1
AG	*AcAG*	Aco009993.1	LG10	2019062–2029924	*OsMADS3*	LOC_Os01g10504.1
					*OsMADS58*	LOC_Os05g11414.1
AGL11	*AcAGL11a*	Aco004785.1	LG05	5785956–5790607	*OsMADS13* *OsMADS21*	LOC_Os12g10540.1 LOC_Os01g66290.1
	*AcAGL11b*	Aco007999.1	LG21	8935775–8941081		
	*AcAGL11c*	Aco011341.1	LG01	12274322–12278849		
SEP1/AGL2/3/4	*AcSEP1*	Aco017563.1	LG09	90797–101510	*OsMADS1*	LOC_Os03g11614.1
					*OsMADS5*	LOC_Os06g06750.1
					*OsMADS34*	LOC_Os03g54170.1
SEP3/AGL9	*AcSEP3*	Aco015105.1	LG01	24291803–24304406	*OsMADS7*	LOC_Os08g41950.1
					*OsMADS8*	LOC_Os09g32948.1
AGL6	*AcAGL6*	Aco015487.1	LG03	12961737–12978045	*OsMADS6*	LOC_Os02g45770.1
					*OsMADS17*	LOC_Os04g49150.1

### Phylogenetic analysis of pineapple MADS-box genes

**Figure 3:**
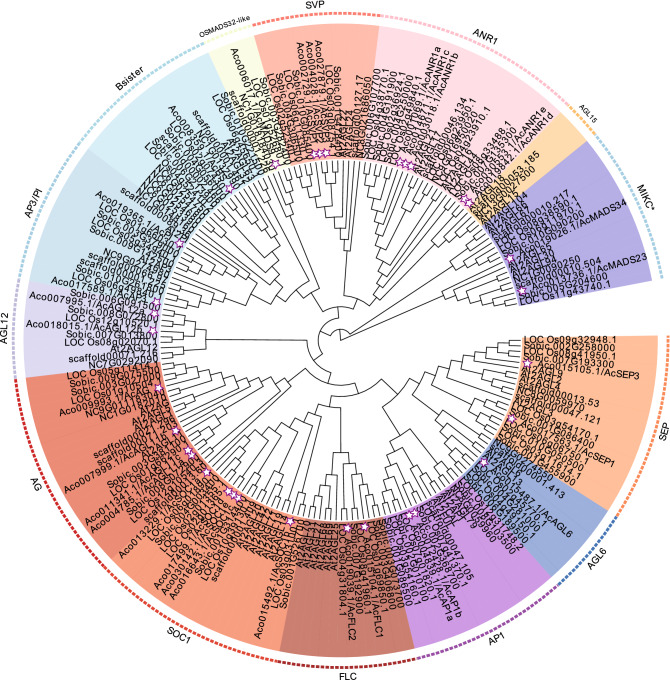
Phylogenetic relationships of type II MIKC^C^ MADS-box proteins in six species. The tree was generated after multiple sequence alignment using MAFFT and the ML method. Fourteen clades are marked with different colors, and the pineapple MADS-box genes are indicated by red dots. The species are abbreviated as follows in the gene names: pineapple, Ac; *Amborella*, Scaffold; waterlily, Nc; *Arabidopsis*, At; rice, Loc_Os; and sorghum, Sobic.

MADS-box genes can be assigned into two phylogenetically distinct groups: type I and type II^28^. To further assess the phylogenetic relationship of pineapple MADS-box genes with those in other species and to assign them to a specific subfamily, two phylogenetic trees were constructed for type I and type II (Fig. 3, Supplementary Fig. S1). Tree construction was based on multiple sequence alignments of MADS-box protein sequences from pineapple and five additional species including basal angiosperm, core eudicot, and monocot species. For the type I group, the 11 pineapple genes were divided into the Mα, Mβ, and Mγ subgroups, with six, two, and three members, respectively, which is similar to other species^6,29^. The type II group are classified into the MIKC^C^ and MIKC* subclades, based on structure divergence at the I domain^30^. The pineapple MIKC^C^ proteins further were divided into 13 subfamilies (AGL6, SEP, AP1/FUL, OSMADS32-like, SOC1, B-sister, AP3/PI, SVP, AGL12, AGL15, ANR1/AGL17, AG/AGL11, and FLC) (Figs. 3, Supplementary Fig. S2). Apart from the AGL15 clade, pineapple MIKC^C^ genes were present in 12 of the 13 clades with their counterparts in *Arabidopsis* and rice, indicating that the genes in the AGL15 clade were lost in pineapple. SOC1 and ANR1/AGL17 were the largest clades, both with five members, whereas AGL6 and OSMADS32-like each had only one member. Compared with the MIKC^C^ gene numbers in rice and orchid, pineapple had fewer members in the AP1, B-PI, Bs, AG, AGL6, and SEP clades. In contrast, significant expansion was observed in the SOC1, ANR1, AGL11, and SVP clades (Fig. 3).

### Conserved ABCDE genes in pineapple

**Figure 4:**
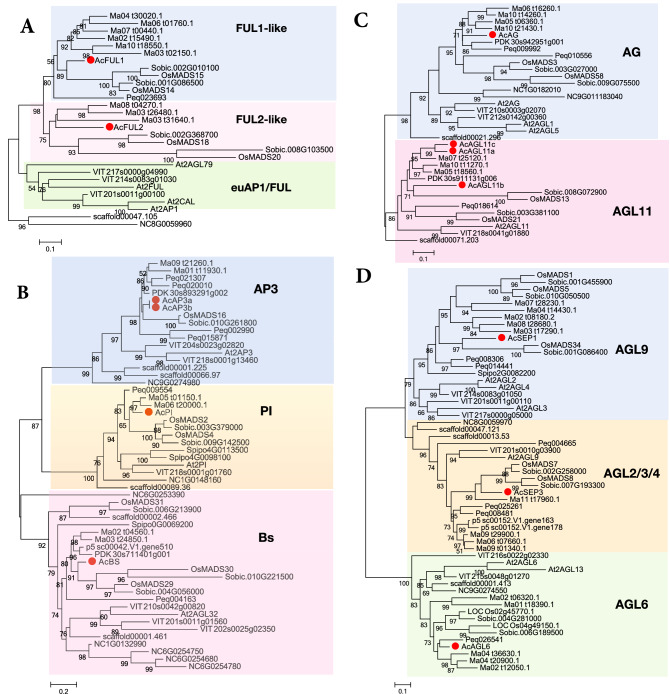
The ML trees of the AP1/FUL-, AP3/PI-, AG/AGL11-, and SEP/AGL6-subfamily genes. The tree was generated after multiple sequence alignment using MAFFT and the ML method. The major clades previously identified in each subfamily are indicated with different colors. The pineapple MADS-box genes are marked by red dots. The species are abbreviated as follows in the gene names: pineapple, Ac; *Amborella*, Scaffold; waterlily, Nc; *Arabidopsis*, At; rice, Loc_Os; and sorghum, Sobic.

We conducted more detailed phylogenetic analyses to identify pineapple genes homologous to *Arabidopsis* and rice genes involved in the ABCDE model of floral development. For A-class genes, the monocot AP1/FUL-like group can be subdivided into two main subclades: FUL-like I and FUL-like II^7^. The two AP1/FUL-like members from pineapple were evenly assigned to these two subclades, with *AcFUL1* closely related to the rice FUL-like I genes *OsMADS15* and *OsMADS14*, and *AcFUL2* similar to the rice FUL-like II genes *OsMADS18* and *OsMADS20* (Fig. 4A). The three B-class candidates were designated as *AcAP3* and *AcPI*. Phylogenetic analysis showed that *AcAP3* belonged to the B-AP3 clade, and only one member, *AcPI*, corresponded to the B-PI clade (Fig. 4B). Because most monocots have only one B-AP3 member, except for orchid with four copies^17,31^(Fig. 4B). *AcBS*, the B-sister homolog in pineapple, formed a sister group with the genes from the AP3/PI clade.

**Figure 5:**
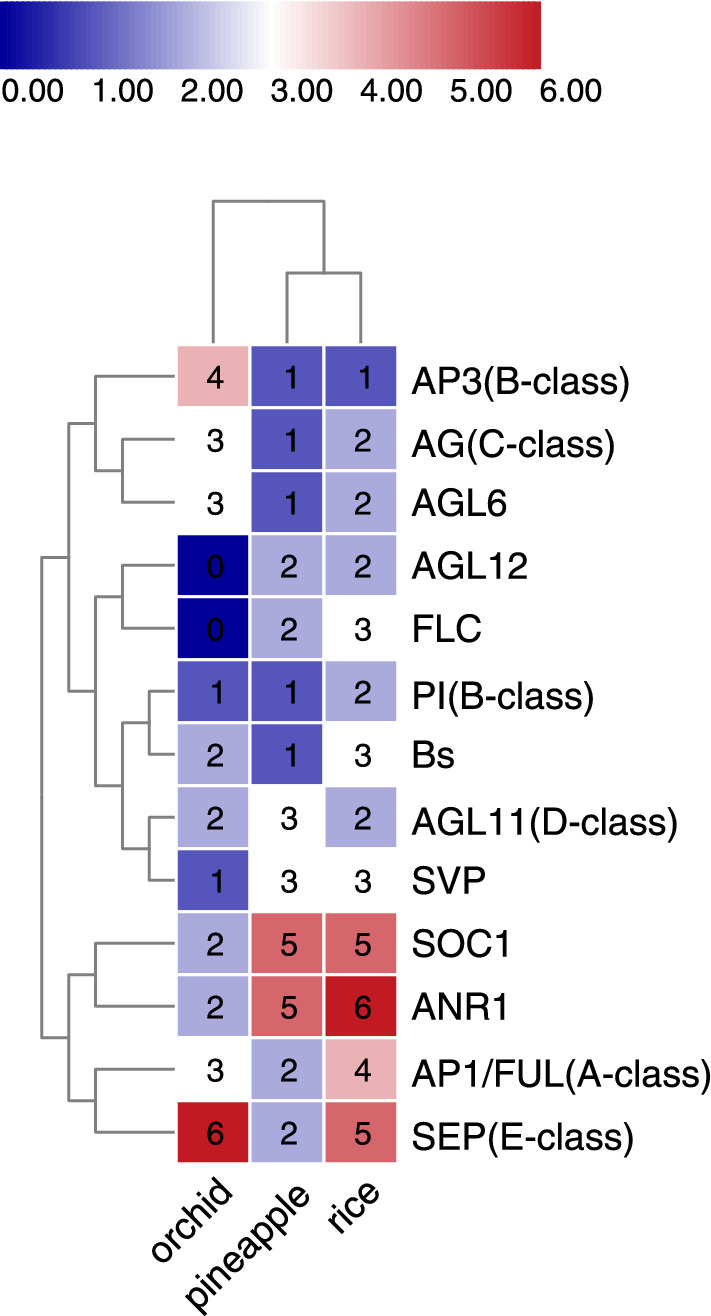
Heat map of the number of MIKC genes from different clades in rice, pineapple, and orchid.

In pineapple, the AG-like subfamily had four members, one of which (*AcAG*) consistently grouped into the C lineage (AG), while the others (*AcAGL11a-c*) were associated with D function (STK/AGL11) (Fig. 4C). According to the sequence and phylogenetic analyses, *AcAG* formed a monophyletic lineage along with two rice MADS-box genes (*OsMADS3* and *OsMADS58*), whereas *AcAGL11a*, *AcAGL11b*, and *AcAGL11c* formed another monophyletic lineage along with the D-lineage MADS-box genes from other monocot species (e.g., *OsMADS13* and *OsMADS21*)*.* The SEP subfamily in pineapple had only two members, *AcSEP1* and *AcSEP3*, compared to five in rice and six in orchid (Fig. 4D). The topology of the phylogenetic trees grouped *AcSEP3* into a subclade (AGL9 or SEP3) with two rice genes, *OsMADS7* and *OsMADS8*. *AcSEP1* was grouped into another subclade (AGL2/3/4 or SEP1) and formed a separate monocot subgroup with three rice MADS-box genes, *OsMADS1*, *OsMADS5*, and *OsMADS34*. There was only one member from pineapple in the AGL6 lineage, compared to two homologous genes in rice, *OsMADS6* and *OsMADS17*. Overall, the phylogenetic analysis revealed that pineapple has fewer ABCE genes compared to rice and orchid (Fig. 5).

### Expression patterns of MADS-box genes during vegetative and reproductive development

**Figure 6:**
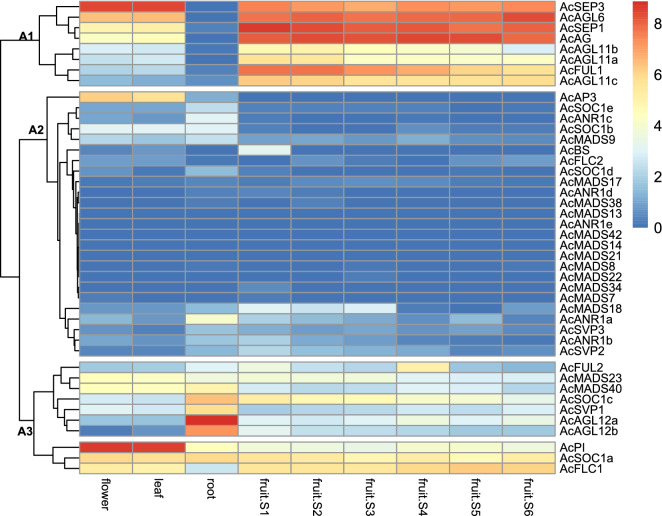
Expression cluster analysis of pineapple MADS-box genes in different tissues and fruit developmental stages. The different tissues and developmental stages included flowers and roots, leaves, and different developmental stages of fruit (S1–S7). The expression value was quantified as fragments per kilobase per million reads (FPKM), and relative gene expression data were gene-wise normalized. Three major expression groups are marked as A1, A2, and A3. The color scale bar is at the top-right corner; blue, white, and red indicate low, medium, and high expression levels, respectively.

Using publicly available transcriptome data and the expression profiles of MADS-box genes in specific developmental processes in pineapple^19,32,33^, we analyzed expression levels in four vegetative and reproductive organs (root, leaf, flower, and fruit) at different developmental stages (Fig. 6; Supplementary Table S2). Three major clusters (A1, A2, and A3) of expression patterns were distinguished according to the expression specificity in different tissues. The A1 cluster included two expression groups. The first group comprised *AcSEP1*, *AcSEP3*, *AcAGL6*, and *AcAG*, which were expressed primarily in leaves and flowers and accumulated at very high levels during fruit development. In the second group, all three members classified as D genes (*AcAGL11a-c*) and one AP1 member (*AcFUL1*) were strictly restricted to fruit. The A2 cluster contained almost all Type I genes (except for *AcMADS40*) and several members from MIKC subclades (e.g., *AcBS*, *AcSOC1d/c*, and *AcSVP1/3*). These genes exhibited relatively low transcript accumulation in almost all tissues except for the B-class genes *AcAP3*, which were moderately expressed in flowers and leaves, and *AcANR1a* expressed in roots. The genes in the A3 cluster segregated into three major expression groups. The first group contained three genes, *AcFUL2*, *AcMADS23*, and *AcMADS40. AcFUL2* was mainly detected in stage S4 fruit, and *AcMADS23* and *AcMADS40* were expressed in flowers, leaves, and roots. The second group comprised *AcSOC1c*, *AcSVP1*, and *AcAGL12a/b*, which had root-specific expression. The third group comprised *AcPI*, *AcSOC1a*, and *AcFLC1*. *AcSOC1a* and *AcFLC1* were evenly expressed in almost all organs, whereas *AcPI* exhibited tissue-specific expression, with FPKM values more than 20-fold higher in flowers and leaves compared to roots.

### Expression patterns of floral MADS-box genes in pineapple

**Figure 7:**
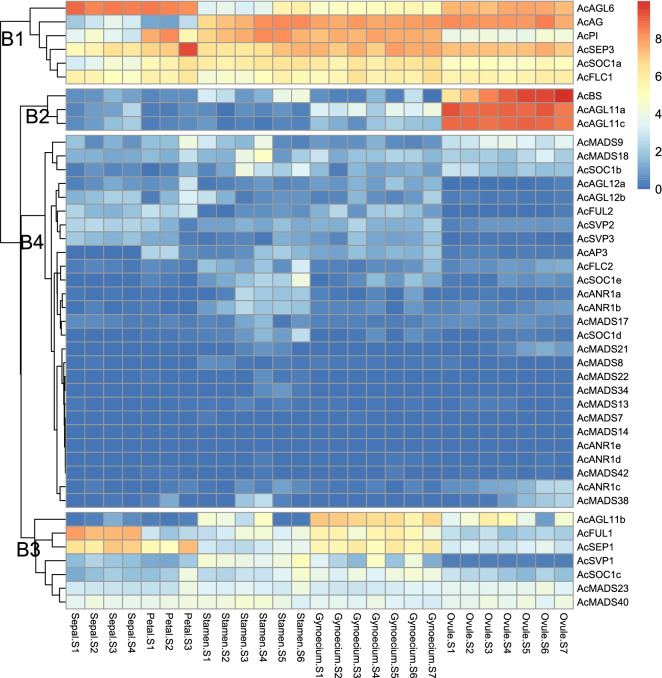
Expression heat map of MADS-box genes in five representative floral organs (sepals, petals, stamens, pistils, and ovules) at different developmental stages of pineapple. The different developmental stage samples comprised four sepal stages (S1–S4), three petal stages (S1–S3), five stamen stages (S1–S5), and seven stages of pistils (S1–S7) and ovules (S1–S7). The expression value was quantified as fragments per kilobase per million reads (FPKM), and relative gene expression data were gene-wise normalized. *AcMADS43* was not detected at any developmental stage. Four major expression groups were marked as B1, B2, B3, and B4. The color scale bar is at the top-right corner; blue, white, and red indicate low, medium, and high expression levels, respectively.

To determine whether certain genes were associated with specific pineapple floral organs, we analyzed RNA-seq transcriptome data for MADS-box genes in five floral organs (sepals, petals, stamens, pistils, and ovules) at different developmental stages^32,33^. Four major expression clusters (B1, B2, B3, and B4) were observed for all MADS-box genes after expression normalization and cluster analysis (Fig. 7; Supplementary Table S3). The B1 cluster included six genes, which were subdivided into two expression groups. The first group contained three genes (*AcAGL6*, *AcAG*, and *AcPI*) that were highly expressed in three floral organs. *AcSEP3*, *AcSOC1a*, and *AcFLC1* in the second group all had abundant transcript levels in all whorls of floral organs, which indicated extensive involvement in the physiological processes of flower development and organogenesis. The B2 cluster contained three genes, a BS subfamily gene (*AcBS*) and two AGL11 subfamily genes (*AcAGL11a* and *AcAGL11c*). These were highly expressed throughout all developmental stages of only ovules. The B3 cluster had three expression groups, and the genes exhibited diverse expression profiles. The first expression group contained three genes, *AcAGL11b*, *AcFUL1*, and *AcSEP1*, which belonged to distinct MIKC subfamilies. *AcAGL11b* was mainly expressed in reproductive organs (stamens and pistils). *AcFUL1* was mostly detected in whorls 1 and 4; *AcSEP1* in whorls 1, 2, and 4. In the second group, *AcMADS23* and *AcMADS40* were detected at low levels in all four whorls. The third group included three genes with moderate or low expression in certain stages of a specific whorl. The remaining MADS-box genes composed the B4 cluster and included most of the type I genes (except for *AcMADS40*) and some members from the SVP, ANR, and SOC1 subfamilies.

Consistent with the expression analysis results in floral tissues, these genes had low transcript levels or no significant expression in each of the floral organs, suggesting that they are not important for floral organ development. *AcMADS43* expression was not detected at any developmental stage. The putative ABCDE model genes had typical temporal and spatial expression profiles in the five analyzed floral organs. The expression of *AcFUL1*, an A-class gene, was high in sepals but was also detected at lower levels in pistils and stamens. The B-PI lineage gene *AcPI* was highly expressed in petals, stamens, and pistil tissues, whereas AP3 lineage gene, *AcAP3*, showed no appreciable expression in these organs. The B-sister class gene *AcBs* was only expressed in ovules, and its transcripts accumulated during the process of ovule development. The C-class gene *AcAG* retained the ancestral function in female and male reproductive organs and was highly expressed in stamens, pistils, and ovules. Interestingly, two D-class genes, *AcAGL11a* and *AGL11c*, showed characteristic expression in ovules suggestive of redundant gene function. However, *AcAGL11b*, also within the D lineage, was expressed in pistils as well as stamens and ovules. Thus, *AcAGL11b* expression was more similar to that of C-class *AcAG* than D-class *AcAGL11a* and *AGL11c*. The E-class genes *AcSEP1* and *AcSEP3* were detected in all floral organs during development. The transcript level of *AcSEP3* was greater than that of *AcSEP1*, indicating that this gene pair might have undergone sub-functionalization after duplication. *AcAGL6* from the AGL6 clade had higher expression levels in sepals, petals, and ovules than in pistils and stamens.

**Figure 8:**
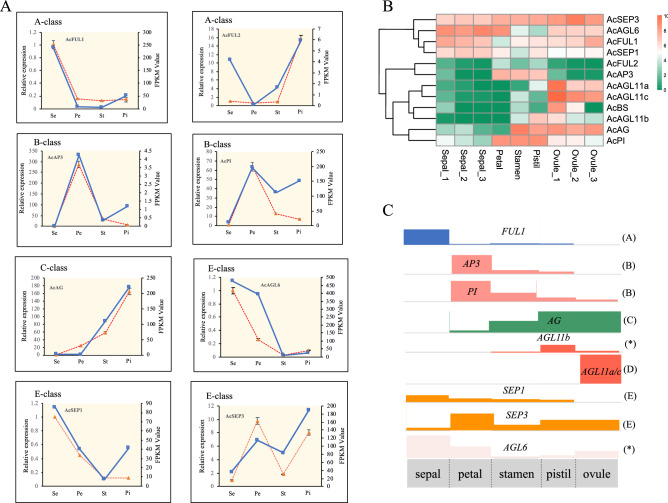
Floral organ-specific expression patterns of AcMADS genes and a deduced pineapple ABCDE model. (**A**)The left y-axis scales the relative expression of RT-qPCR result and Blue solid line indicated values of RT-qPCR.The right y-axis scales the FPKM value from RNA-seq result(stage1 sepal, petal, stamen, pistil) and orange dashed line indicated values of RT-qPCR. (**B**) Heatmap plot of expression patterns of pineapple A-, B-, C-, D-, E-class genes from another pineapple tissue-specific transcriptome. (**C**) A proposed pineapple flower model based on the expression patterns and referring to the ancestral functions of homeotic MADS-box genes. Blocks in different colors with distinct heights represent different expression levels. Gene functions for specific members are in brackets. *The gene function is difficult to predict because an expression split has occurred. The gaps are linked with grey lines.

Even though RNA-seq data had been proved reliable by RT-qPCR and in situ hybridization, we selected eight A, B, C, E class orthologous genes (*AcFUL1, AcFUL2, AcAP3, AcPI, AcAG, AcAGL6, AcSEP1, AcSEP3*) to test their expression in four whorls of floral organs (sepal, petal, stamen, pistil) by RT-qPCR in pineapple. The RT-qPCR results were consistent highly with our RNA-seq results (Fig. 8A) and another published dataset of pineapple tissue-specific transcriptome^34^ (Fig. 8B), which provided solid proofs for the subsequent propose of ABCDE gene model (Fig. 8C).

## Discussion

Our bioinformatics analysis identified 43 MADS-box genes in the pineapple genome. The phylogenetic analysis divided the type II MADS-box genes into 13 subfamilies, among which the OsMADS32-like clade appeared to be a novel monocot-specific lineage^29,35,36^. Two members from the FLC clade were identified in the *A. comosus* genome, but their relationship to genes from other species, such as *Arabidopsis*, was not well resolved. The low support value might be due to the highly divergent sequences and extremely short length of monocot *FLC* genes^37^. The gene numbers in most MIKC subfamilies were less than those identified in rice and sorghum, consistent with the idea that grasses underwent a recent whole genome duplication (WGD) after the divergence from the Bromeliaceae^19^. The reduced number of MADS-box gene copies in pineapple has important implications for elucidating the evolutionary history of the MADS-box gene family prior to the divergence of grasses and pineapple.

### Different evolutionary patterns of MADS-box genes in pineapple and rice

The phylogenetic analysis revealed two A-class, three B-class, one C-class, three D-class, and two E-class homologs in pineapple (Table 1). Although pineapple and rice shared a WGD event near the base of monocot plants, only one pair of duplicated genes, *AcFUL1* and *AcFUL2*, was retained. Another gene pair, *AcAGL11a and AcAGL11c*, probably arose from species-specific duplication in pineapple. Unsurprisingly, no gene species-specific duplication was found in pineapple within AP3/PI lineage. Indeed, in most monocots, only one B-AP3 and B-PI copy has been reported^17^. Therefore, gene duplication in this subclade is rare, except in orchids where gene duplication events occurred frequently, followed by expression divergence that led to the innovation of specialized labellum^38^. Rice had at least seven pairs of genes in the AP1, PI, AG, AGL, AGL2/3/4, AGL9, and AGL6 lineages that derived from a more recent WGD event that occurred prior to the origin of the Poaceae 66–70 MYA^17,39^. The significant expansion of genes in the B-AP3 and E classes in orchid may have also resulted from the WGD^38^. In contrast, there were fewer instances of recent gene duplications in pineapple, showing that the composition of ABCDE genes in pineapple was conserved and that the reduced gene numbers in these model classes are representative of an ancestral state. The five members in the SOC1 clade, five members in the ANR1 clade, and three members in the SVP clade suggest that these subfamilies expanded frequently (Fig. 5). Thus, contraction of the gap between the number of type II genes in pineapple (31) and rice (43) may have been a consequence of the expansion of these clades. Among them, the expression profiles of SOC1 subfamily genes were diverse, with each member having a distinct expression pattern. Because *SOC1* genes are primarily involved in the flowering phase transition and stress tolerance^40,41^, the expansion and diversification of the SOC1 subfamily in pineapple may have contributed to its adaptation to extreme tropic environments.

### Pineapple MADS-box genes involved in fruit development

In most cases, MADS-box genes within the same phylogenetic subgroup exhibited analogous expression patterns, indicating that these genes have similar biological functions in shaping regulatory networks that influence specific developmental processes. The genes clustered in the AP3/PI, AG, and SEP/AGL6 clades were highly expressed in pineapple reproductive organs, consistent with their expected roles in floral organogenesis and development. Notably, most of the genes belonging to the AP1/FUL (*AcFUL1*), AG/AGL11 (*AcAG*, *AcAGL11a/b/c*), SEP (*AcSEP1/3*), and AGL6 (*AcAGL6*) subfamilies in pineapple were highly expressed throughout all fruit developmental stages. In tomato, a fleshy fruit model plant, several genes including *SlMADS-RIN* from the SEP subfamily, *TAGL1* from the AG-like subfamily, and *FUL1* and *FUL2* from the AP1-like subfamily regulate both early fruit expansion and later ripening^42–45^. In a study in banana, two SEP-like subfamily genes, *MaMADS1* and *MaMADS2*, were functionally characterized, and repression of either gene led to slow-ripening and prolonged shelf-life phenotypes^46^. The present finding that *AcFUL1* transcript levels were relatively high in fruits is consistent with the hypothesis that the AP1/FUL clade in monocots includes only *FUL*-like rather than *AP1*-like sequences, and that these genes may control fruit development analogously to the *Arabidopsis* gene *FRUITFULL*^47^. In summary, MADS-box genes from these subfamilies may play a conserved and essential role during fruit development and the ripening of the non-climacteric fleshy fruit of pineapple and therefore warrant further study in the context of pineapple fruit yield and storage.

Unlike the ABCDE genes of *Arabidopsis* and *Antirrhinum* whose expression is limited to flowers, many B-, C-, and E-class pineapple genes were also expressed in leaves. For example, *AcSEP1* and *AcPI* were up-regulated in flowers and highly expressed in leaves, suggesting that these genes may also be critical for vegetative development. *AcAGL12a* and *AcAGL12b* had preferentially high expression levels in roots, with sparse expression in leaves and fruits, indicating that these two genes are root-specific and may be important for root development (Fig. 6). Almost all type I MADS-box genes had relatively low transcript levels or no significant expression based on their FPKM values from the RNA-seq data for different tissues. This finding is consistent with earlier reports that type I genes have relatively limited functions in plants compared to type II genes^2,48^.

### Functional conservation of ABCDE model genes in pineapple

The expression of A-class *AcFUL1* in pineapple was notably higher in sepals than in other floral tissues, which is in line with its expected function in sepal identity specification. However, its expression was extremely low in petals, indicating that other A-function genes might be recruited to specify sepal identity in pineapple. *AcFUL1* was also detected at moderate levels in pistils, a pattern also reported in orchid, in which two AP1/FUL-like members, *PhaMADS1* and *PhaMADS2*, promote carpel and ovary development before and after pollination^31,49^. There is evidence from a study of water lily and *Nigella damascena* that the role of AGL6-like subfamily genes is similar to that of *AP1*^50^. In orchid, these genes have a specialized function in labellum formation^49^. Although *AcAGL6* was expressed in all tissues in our study, its expression was highest in sepals and petals, indicating a possible role in the A-class functions of whorl 1 and 2 specification via interaction with *AP1* and *SEP*.

Robust expression levels of *AcAP3* and *AcPI* expression in petals and stamens indicated that these two genes performed B function based on the conserved expression patterns of B-class genes in angiosperms^51^. The Bs subgroup, a phylogenetic sister group of the B-class floral homeotic genes, is specifically expressed in female reproductive organs and developing seeds^52,53^. Both the sequence and ovule-specific expression profile of pineapple *AcBs* were highly similar to those of the rice ortholog *OsMADS29*, whose expression is restricted to developing seeds^52^. In line with ancestral C function in specifying both male and female reproductive organs, high expression levels of *AcAG* were detected in stamens, pistils, and ovules (Fig. 8a). Regarding D function, three subfamily members (*AcAGL11a*, *AcAGL11b*, and *AcAGL11c*) were detected and found to be homologous to orchid *MADS2* and rice *OsMADS13/21*^54,55^. *AcAGL11a/c* were identified as D-function candidate genes because their expression patterns in our study were similar to those of other D-lineage genes, which are preferentially expressed in ovules. The expression of *AcAGL11b* in the inner whorls of flowers and ovules overlapped with the expression domain of *AcAG*. In rice, *OsMADS21* exhibits the same expression pattern but lost its function in determining ovule identity, presumably because of its redundancy with *OsMADS13*, whose expression is also restricted to the female reproductive organs^15^. We therefore hypothesized that like *OsMADS21*, *AcAGL11b* might have similarly lost its role in D function. *AcSEP3* is more likely to have a major role in E function because it had higher transcript levels than *AcSEP1* in all organs except sepals. Additionally, *AcSEP3* orthologs, including *AtAGL9* in *Arabidopsis* and *OsMADS7/8* in rice, are more critical for E function than any other SEP-like family members in the two plants^56,57^. By comparing the expression patterns of pineapple MADS-box genes with those of previously characterized orthologs, we inferred the functions of candidate pineapple genes involved in the ABCDE model. The numbers and evolutionary history of the putative ABCDE genes strongly indicated that the pineapple flower is similar to the ancestral state of monocot flowers.

## Materials and methods

### Data sources and sequence retrieval

The whole pineapple genome sequences that were used to identify MADS-box genes were downloaded from the Pineapple Genomics Database^58^. Additionally, the water lily (*Nymphaea colorata*) genome was generated from our own genome project^59^. The MADS-box protein sequences of *Arabidopsis* and rice were retrieved from the TAIR (http://www.arabidopsis.org/) and RGAP (http://rice.plantbiology.msu.edu/) databases, respectively. Non-redundant protein sequences of *Amborella trichopoda*, *Vitis vinifera*, *Sorghum bicolor*, *Musa acuminate*, and *Spirodela polyrhiza* were collected from Phytozome (http://www.phytozome.net/). The latest proteome release of *Phalaenopsis equestris* was from a recent study^38^. The proteome of *Elaeis guineensis* was downloaded from the Genomsawit website (http://genomsawit.mpob.gov.my/index.php?track=30), and the *Phoenix dactylifera* proteome was obtained from the Date Palm Research Program (http://qatar-weill.cornell.edu/research/research-highlights/date-palm-research-program).

### Genome-wide identification of MADS-box genes

To identify MADS-box gene family members in pineapple, the hidden Markov model (HMMER) profile of the SRF-TF domain (Pfam accession: PF00319) was obtained from the Pfam database (http://pfam.xfam.org/)^60^ used as a query to search against pineapple proteins. The gene IDs and sequence information are provided in Table 1 and Supplementary Table S1. In addition to pineapple, MADS-box protein sequences in the following species were also collected and screened to investigate their evolutionary relationships: two basal angiosperm species (*Amborella* and waterlily); two core eudicots (*Arabidopsis* and *Vitis vinifera*); and seven monocot species (rice, sorghum, *Phalaenopsis equestris* (Epidendroideae), *Musa acuminata* (Musaceae), *Elaeis guineensis* (Arecaceae), *Phoenix dactylifera* (Arecaceae), and *Spirodela polyrhiza* (Lemnoideae)).

### Classification of MADS-box genes in pineapple

To assign putative pineapple MADS-box genes to specific gene subfamilies, multiple sequence alignments were performed based on amino acid sequences using the alignment tool MAFFT with default parameter settings^61^. MADS-box proteins from six plant species, *A. trichopoda*, *N. colorata*, *A. thaliana*, *O. sativa*, *S. bicolor*, and *A. comosus*, were used. Maximum-likelihood phylogenetic trees were constructed using FastTree software with the JTT+CAT model^62^. Furthermore, a more detailed MIKC-protein phylogenetic tree was constructed using the same strategy with six additional species: *V. vinifera* (Vitaceae), *P. equestris* (Epidendroideae), *M. acuminate* (Musaceae), *E. guineensis* and *P. dactylifera* (Arecaceae), and *S. polyrhiza* (Lemnoideae). In the phylogenetic tree, bootstrap supporting values below 50 were generally regarded as unreliable and were not shown. As a matter of convenience, pineapple MIKC^C^ genes were renamed according to the phylogenetic relationship deduced by sequence comparison with proteins from the whole genomes of 11 flowering plants and their corresponding clade/subclade names. A phylogenetic tree of pineapple and ten other species (Fig. 2) was also constructed in the context of angiosperms. The phylogeny was inferred using RAxML v7.1.0 with the PROTGAMMAJTT model, 100 bootstrap replicates, 756 single-copy genes, and the methods described above^63^.

### Expression profiles of MADS-box genes in different pineapple tissues

Two transcriptome datasets were extracted from previous studies in pineapple^19,32^. The first dataset included floral, leaf, and root developmental tissues and six stages of fruit development (F1–F6, from young to mature)^19^. The other dataset was composed of different floral organ samples, including four sepal stages (S1–S4), three petal stages (S1–S3), five stamen stages (S1–S5), seven pistil stages (S1–S7), and seven ovule stages (S1–S7). The criteria for the different stages were previously described in details^32,33,64^. The expression level of each gene was quantified as fragments per kilobase of exon model per million reads mapped (FPKM) values by featureCounts^65^. The third dataset from previous published study provided available expression matrix including different floral organs in transcripts per kilobase million (TPM) by Stringtie^34,66^. Two heat expression maps were generated by the Pheatmap package in R software using pre-treated FPKM values by log^2^ transformation. Differences in gene expression levels were represented using a color scale.

### RNA isolation and RT-qPCR

Total RNA was extracted from tissues following manufacturer’s guidelines RNA extraction Kit (Omega Bio-Tek, Shanghai, China). cDNA was synthesized from 1 μg of RNA using the EasyScript One-Step gDNA Removal and cDNA Synthesis SuperMix (Transgen, Beijing, China). RT-qPCR analysis was carried out using TransStart Top Green qPCR SuperMix (Transgen, Beijing, China). The primers of MADS-box genes and internal control (*Actin2*) used for RT-qPCR were shown in Supplementary Table S5. To confirm results reliability, three biological replicates were conducted. 2-ΔCT method was applied to calculate the relative expression of pineapple MIKC^C^ genes. For statistical analysis, normalized relative values are given as mean value ± standard deviations.

## Supplementary Information

Supplementary Information
