# Supplementary material for: The pineapple MADS-box gene family and the evolution of early monocot flower
Source: Sci Rep. 2021 Jan 13;11:849. doi: 10.1038/s41598-020-79163-8 (PMC7806820; doi:10.1038/s41598-020-79163-8)
Supplement: Supplementary file 1 — Supplementary Information [file 41598_2020_79163_MOESM1_ESM.pdf]

SUPPORTING INFORMATION

The pineapple MADS-box gene family and the evolution of early monocot flower

Juan Hu[1], Xiaojun Chang[1], Ying Zhang[1], Xianxian Yu[2], Yuan Qin[1], Yun Sun[1], Liangsheng Zhang[1, 3*]

[1]Fujian Provincial Key Laboratory of Haixia Applied Plant Systems Biology, College of Horticulture, Fujian Agriculture and Forestry University, Fuzhou 350002, China
[2]School of Urban-Rural Planning and Landscape Architecture, Xuchang University, Xuchang 461000, China
[3]Genomics and Genetic Engineering Laboratory of Ornamental Plants, College of Agriculture and Biotechnology, Zhejiang University, Hangzhou 310058, China.

[*]Corresponding author:

Liangsheng Zhang, E-mail: zls83@zju.edu.cn

Running title: The pineapple MADS-box gene family

SUPPORTING INFORMATION

Figure S1. Phylogenetic relationships of type I MADS-box genes in pineapple (Ac), *Amborella* (Scaffold), waterlily (Nc), *Arabidopsis* (At), rice (Loc_Os), and sorghum (Sobic).

Figure S2. Phylogenetic relationships of type II MADS-box genes in the 12 following species: pineapple (Ac), *Amborella* (Scaffold), waterlily (Nc), *Arabidopsis* (At), *Vitis vinifera* (Vt), rice (Loc_Os), sorghum (Sobic), *P. equestris* (Peq), *M. acuminata* (Ma), *E. guineensis* (p5), *P. dactylifera* (PDK), and *S. polyrhiza* (Spipo).

Table S1. List of other pineapple MADS-box genes not included in Table 1.

Table S2. List of FPKM values of all MADS-box genes in flowers and roots, leaves, and different developmental stages of fruit (S1–S7).

Table S3. List of FPKM values of all MADS-box genes in five representative pineapple floral organs (sepals (S1-S4), petals (S1-S3), stamens (S1-S6), pistils (S1-S7), and ovules (S1-S7)) at different developmental stages.

Table S4. List of TPM value of ABCDE model genes in four representative pineapple floral parts (sepal (S1-S3), petal, stamen, pistil and ovule (S1-S3)) at different developmental stages.

Table S5. The primers used in this study.

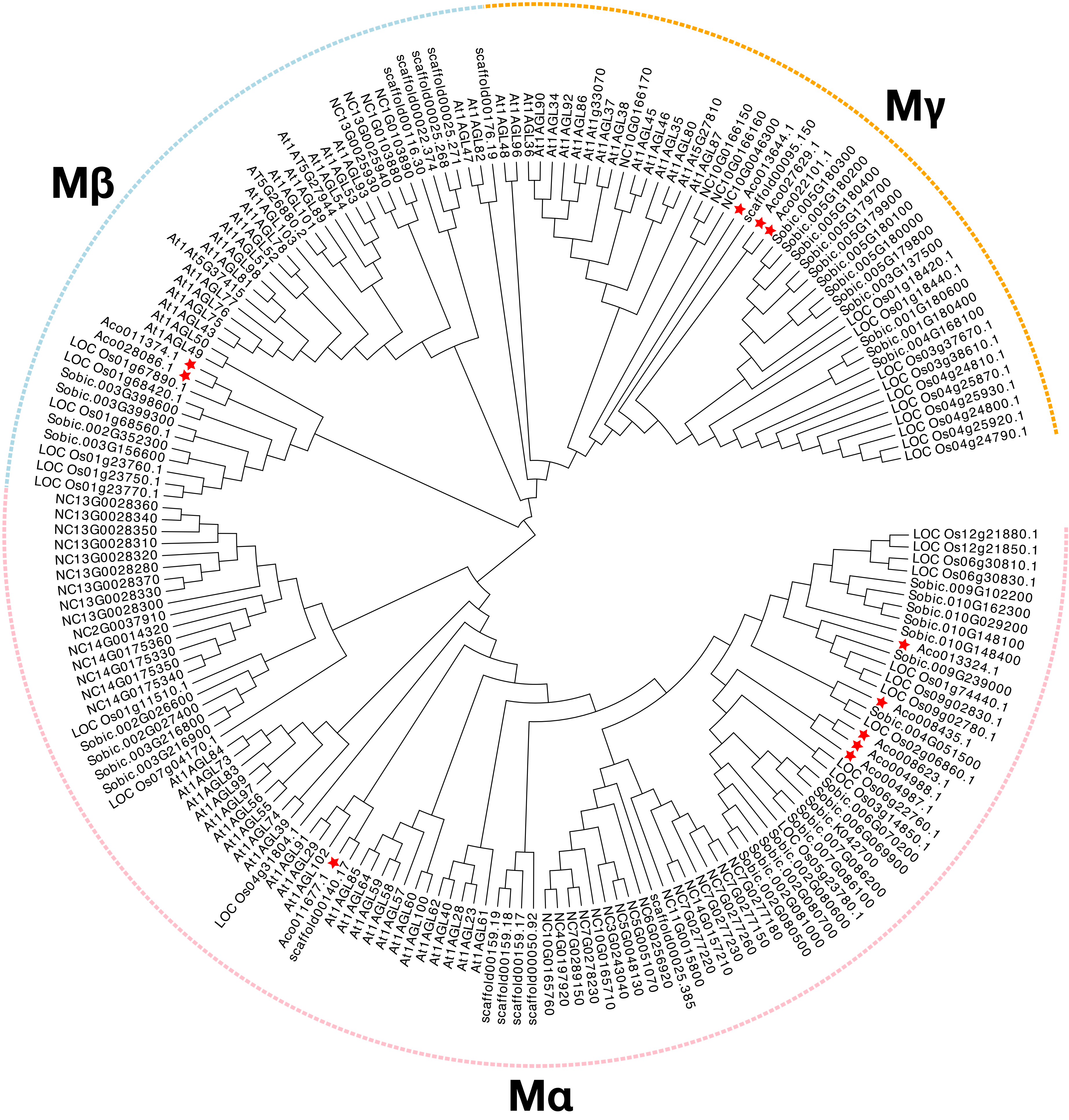
Mβ
Mγ
Mα

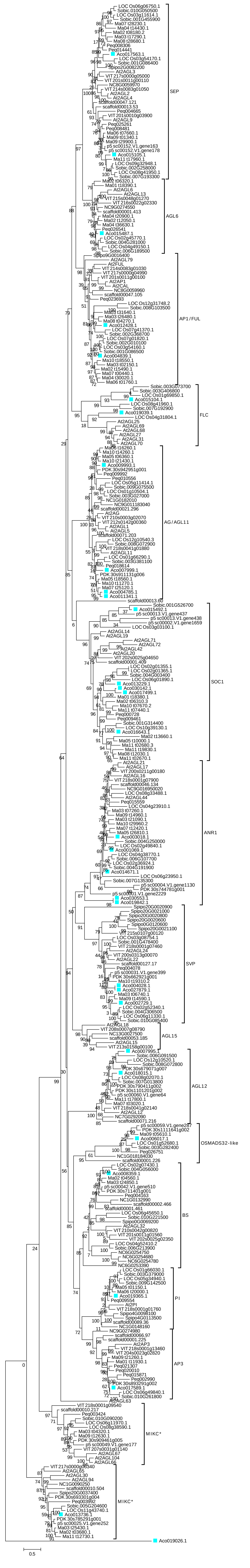

SEP
AGL6
AP1 / FUL
FLC
AG / AGL11
SOC1
ANR1
SVP
AGL15
AGL12
OSMADS32-like
BS
PI
AP3
MIKC*
MIKC*
0.5

Table S1. List of the other MADS-box genes in pineapple besides Table 1.

Subfamily	Gene Name	Gene ID	Chromosome location	
SOC1	AcSOC1a	Aco013229.1	LG24	734598-748716
	AcSOC1b	Aco016643.1	LG08	10668008-10681245
	AcSOC1c	Aco015492.1	LG03	12918841-12919719
	AcSOC1d	Aco030142.1	LG22	1009597-1024390
	AcSOC1e	Aco017499.1	LG22	1357314-1364201
SVP	AcSVP1	Aco002729.1	LG06	11053591-11061483
	AcSVP2	Aco004028.1	LG15	1900301-1908029
	AcSVP3	Aco027879.1	scaffold_1163	8888-16651
ANR1	AcANR1a	Aco003018.1	LG06	13262206-13272758
	AcANR1b	Aco001069.1	LG02	14452429-14467956
	AcANR1c	Aco014671.1	LG20	9897052-9905261
	AcANR1d	Aco019842.1	LG15	9145194-9148873
	AcANR1e	Aco030553.1	scaffold_1319	33959-4138
AGL12	AcAGL12a	Aco007995.1	LG21	8966391-8969977
	AcAGL12b	Aco018015.1	LG01	20412367-20420233
FLC	AcFLC1	Aco015104.1	LG01	24318710-24340976
	AcFLC2	Aco019039.1	LG20	404283-409016
OsMADS32- like	AcMADS9	Aco006017.1	LG16	9688836-9690655
MIKC*	AcMADS23	Aco013736.1	LG13	10739290-10744427
	AcMADS34	Aco019026.1	LG02	11042145-11045945
Mα	AcMADS7	Aco004987.1	LG07	1255014-1255685
	AcMADS8	Aco004988.1	LG07	1256809-1258119
	AcMADS13	Aco008435.1	LG19	10342859-10343854
	AcMADS14	Aco008623.1	LG09	1164861-1165547
	AcMADS18	Aco011677.1	LG08	12300067-12300639
	AcMADS21	Aco013324.1	LG15	11241110-11241826
Mβ	AcMADS17	Aco011374.1	LG01	12049800-12051479
	AcMADS42	Aco028086.1	scaffold_1517	21796-23778
Mγ	AcMADS22	Aco013644.1	LG13	11508561-11509322
	AcMADS38	Aco022101.1	LG04	74921-75724
	AcMADS40	Aco027629.1	scaffold_622	55034-83846

Table S2. List of FPRM value of all MADS-box genes in flower and root, leaf, and different developmental stages of fruit (S1–S6).

	flower	leaf	root	fruit.S1	fruit.S2	fruit.S3	fruit.S4	fruit.S5	fruit.S6
AcSOC1a	63.8	55.6	63.5	48.3	40.8	28.1	38.2	24.6	38.2
AcSOC1e	1.0	1.0	4.1	0.2	0.2	0.2	0.3	0.0	0.1
AcSOC1d	0.6	0.1	1.7	0.2	0.0	0.0	0.0	0.1	0.0
AcSOC1c	5.9	4.8	86.2	38.2	26.3	23.7	15.9	14.5	8.8
AcSOC1b	7.2	7.1	6.8	0.3	0.0	0.0	0.5	0.1	0.2
AcFUL1	3.2	3.7	0.2	212.9	220.1	145.0	111.5	67.1	50.8
AcFUL2	2.4	2.8	6.6	11.3	4.3	3.5	33.5	2.4	1.6
AcAGL6	83.5	88.9	0.0	213.0	267.4	214.2	247.4	239.1	328.6
AcSEP3	345.5	345.9	0.0	183.4	141.6	108.6	154.2	139.0	166.1
AcSEP1	30.0	36.1	0.2	399.3	342.2	276.0	299.1	216.7	252.7
AcAG	18.8	22.7	0.0	275.7	332.5	318.2	350.6	339.0	244.2
AcAGL11b	6.5	5.7	0.1	26.2	30.9	20.8	17.4	14.3	6.5
AcAGL11c	1.7	1.3	0.4	76.0	68.6	50.2	49.3	53.3	58.6
AcAGL11a	4.3	5.6	0.0	51.9	45.7	19.2	18.3	17.3	18.6
AcANR1b	1.0	0.6	2.3	3.0	1.1	0.8	0.3	0.2	0.1
AcANR1a	1.7	0.7	15.5	3.0	1.7	1.2	0.5	1.8	0.3
AcANR1c	1.0	0.7	7.6	0.2	0.0	0.0	0.1	0.0	0.0
AcAGL12a	2.2	2.2	468.0	7.3	5.6	7.4	10.8	6.9	9.7
AcAGL12b	0.2	0.6	145.0	7.6	4.5	4.2	3.6	2.5	2.6
AcBS	0.3	0.7	0.0	7.9	0.0	0.0	0.0	0.0	0.0
AcAP3	69.7	51.5	1.2	0.0	0.0	0.0	0.0	0.0	0.0
AcPI	404.7	379.4	21.3	12.3	10.9	9.7	15.9	15.2	12.0
AcSVP1	7.2	5.7	58.0	2.7	3.6	3.9	5.5	4.1	6.5
AcSVP2	0.4	0.3	1.6	3.6	1.9	1.4	1.2	0.3	0.5
AcSVP3	0.6	0.2	2.1	1.3	0.8	1.1	0.6	1.0	0.4
AcMADS9	3.5	2.4	4.5	0.9	1.1	0.7	1.3	0.4	0.4
AcMADS34	0.0	0.0	0.0	0.4	0.0	0.0	0.0	0.0	0.0
AcMADS23	20.7	19.3	11.9	13.9	12.3	12.3	7.2	6.4	6.6
AcMADS7	0.2	0.0	0.0	0.2	0.0	0.0	0.0	0.0	0.0
AcMADS8	0.0	0.0	0.1	0.0	0.0	0.1	0.0	0.0	0.1
AcMADS13	0.0	0.0	0.0	0.0	0.0	0.0	0.0	0.0	0.0
AcMADS14	0.0	0.0	0.0	0.0	0.0	0.0	0.0	0.0	0.0
AcMADS17	0.1	0.2	0.3	0.2	0.4	0.5	0.4	0.2	0.1
AcMADS18	0.7	0.7	1.7	7.3	4.9	6.5	0.2	0.0	0.8
AcMADS21	0.0	0.0	0.0	0.0	0.0	0.0	0.0	0.0	0.0
AcMADS22	0.0	0.0	0.0	0.0	0.0	0.2	0.0	0.0	0.0
AcFLC1	38.7	31.8	5.1	45.8	45.2	42.8	64.4	73.7	68.5
AcFLC2	0.8	0.8	0.1	0.0	0.6	0.2	0.0	0.6	0.8
AcANR1d	0.0	0.0	0.3	0.3	0.1	0.0	0.0	0.0	0.0
AcMADS38	0.0	0.0	0.2	0.0	0.2	0.0	0.0	0.0	0.0
AcMADS40	21.6	19.5	28.6	6.0	3.7	4.3	7.2	6.4	3.6
AcANR1e	0.0	0.0	0.0	0.0	0.0	0.0	0.0	0.0	0.0

Table S3. List of FPKM value of all MADS-box genes in five representative pineapple floral parts (sepal (S1-S4), petal (S1-S3), stamen (S1-S6), carpel (S1-S7), and ovule (S1-S7)) at different developmental stages

	Sepal.S	Sepal.S	Sepal.S	Sepal.S	Petal.S	Petal.S	Petal.S	Stamer	Stamer	Stamer	Stamer	Stamer	Stamer	Pistil.S	Pistil.S	Pistil.S	Pistil.S	Pistil.S	Pistil.S	Pistil.S	Ovule.S	Ovule.S	Ovule.S	Ovule.S	Ovule.S	Ovule.S	Ovule.S7
AcANR1a	0.1	0.0	0.1	0.0	0.1	0.1	0.0	0.1	0.3	5.0	3.2	2.6	3.2	0.3	0.1	0.0	0.4	0.0	1.1	0.1	0.1	0.1	0.2	0.1	0.1	0.1	0.2
AcSVP1	2.1	4.1	3.0	2.5	1.4	1.2	3.2	20.5	16.2	13.5	11.3	17.0	35.0	9.9	4.2	1.9	26.8	2.5	16.8	1.6	0.2	0.2	0.2	0.2	0.3	0.2	0.2
AcANR1b	0.0	0.3	0.1	0.1	0.2	0.0	0.0	1.0	1.9	4.9	2.9	3.1	2.6	0.3	0.4	0.4	0.5	0.1	0.8	0.3	0.4	0.6	0.4	0.3	0.4	0.8	0.9
AcSVP2	4.2	5.0	3.7	4.2	3.6	1.8	0.3	1.1	1.1	1.8	1.2	2.3	1.3	1.8	1.6	4.0	1.8	1.5	1.8	3.9	0.3	0.4	0.9	0.8	0.8	0.6	0.9
AcAGL11a	0.4	0.9	1.0	4.6	0.2	0.2	0.5	0.5	0.0	0.5	0.4	0.6	0.3	8.7	6.5	11.6	3.3	12.3	8.7	15.6	661.2	483.9	491.8	531.6	506.1	620.6	385.9
AcFUL1	239.8	161.9	177.5	188.4	6.9	3.0	4.8	5.3	7.9	18.1	19.5	8.6	16.0	50.0	48.1	19.7	46.4	60.7	41.0	17.0	5.8	3.4	5.8	2.4	3.5	4.4	3.4
AcMADS7	0.0	0.0	0.0	0.0	0.0	0.0	0.0	0.2	0.0	0.0	0.0	0.0	0.0	0.0	0.0	0.1	0.0	0.0	0.0	0.0	0.0	0.0	0.0	0.0	0.0	0.0	0.0
AcMADS8	0.0	0.1	0.0	0.0	0.0	0.1	0.0	0.5	0.5	0.1	0.0	0.2	0.1	0.2	0.1	0.1	0.1	0.0	0.1	0.1	0.2	0.1	0.1	0.1	0.0	0.1	0.1
AcMADS9	2.5	0.5	1.8	0.6	2.3	2.4	15.6	7.2	2.6	1.9	14.0	0.5	0.3	0.8	0.6	0.6	1.6	0.4	0.4	0.7	5.8	6.6	11.8	12.0	9.2	5.9	10.3
AcAGL12a	0.3	0.6	1.4	0.8	0.6	0.8	6.8	0.1	0.3	0.4	0.8	0.4	0.4	0.9	0.7	1.7	0.9	2.8	1.2	2.0	0.0	0.0	0.0	0.0	0.0	0.1	0.2
AcAGL11b	0.1	0.3	1.6	0.4	0.2	0.1	0.2	21.5	15.8	5.4	27.8	0.1	0.1	143.0	130.5	113.8	97.7	97.6	46.4	106.2	10.7	20.5	57.2	37.5	13.7	0.9	20.5
AcBS	0.5	0.7	1.8	1.1	0.4	0.5	0.6	7.9	5.1	0.8	1.6	13.4	17.1	0.6	0.3	0.3	2.0	0.3	5.9	0.0	75.2	162.4	341.7	563.6	751.9	751.5	#####
AcMADS13	0.1	0.0	0.0	0.0	0.0	0.0	0.1	0.1	0.1	0.3	0.1	0.3	0.1	0.1	0.1	0.0	0.1	0.0	0.1	0.1	0.0	0.2	0.2	0.2	0.0	0.1	0.1
AcMADS14	0.0	0.0	0.0	0.0	0.0	0.0	0.0	0.0	0.0	0.1	0.0	0.0	0.0	0.0	0.0	0.0	0.0	0.0	0.0	0.0	0.0	0.0	0.0	0.0	0.0	0.0	0.0
AcAG	3.1	6.7	12.8	7.7	1.2	1.0	5.1	109.7	153.6	180.5	294.5	315.2	334.7	221.7	203.3	255.0	221.1	222.2	251.9	254.4	318.7	290.2	259.6	275.2	268.1	385.6	198.7
AcAGL11c	0.4	0.9	2.3	3.4	0.2	0.4	0.5	0.8	0.1	0.3	0.1	0.4	0.3	2.5	1.5	0.6	2.8	3.8	2.5	2.3	501.7	503.9	428.0	493.0	520.8	477.6	393.4
AcMADS17	0.5	0.4	0.3	0.2	0.2	0.3	0.2	0.4	0.3	1.1	2.2	0.1	0.6	0.2	0.3	0.2	0.2	0.2	0.2	0.5	0.5	0.6	0.4	0.7	0.6	0.5	0.4
AcMADS18	1.8	1.8	1.2	1.3	2.2	1.5	5.6	1.9	1.8	13.1	28.5	0.8	3.6	6.4	1.4	2.7	2.5	3.9	3.9	1.9	3.8	3.2	3.4	2.3	4.8	8.6	3.5
AcFUL2	4.2	1.9	1.8	2.6	6.0	3.8	6.8	0.1	0.0	0.6	0.6	0.8	0.6	1.7	4.3	0.6	3.3	3.8	2.3	1.0	0.0	0.0	0.1	0.1	0.0	0.0	0.2
AcSOC1a	8.1	11.9	21.0	16.6	38.9	47.4	84.4	61.1	48.2	58.9	77.9	81.0	84.4	68.5	47.1	76.0	53.2	81.2	79.7	95.9	28.7	33.1	33.8	26.8	28.4	41.4	31.2
AcMADS21	0.0	0.0	0.0	0.0	0.0	0.0	0.0	0.0	0.0	0.3	0.7	0.5	0.0	0.0	0.0	0.0	0.0	0.0	0.0	0.0	0.1	0.1	0.1	0.4	1.1	1.6	0.9
AcMADS22	0.0	0.0	0.0	0.0	0.1	0.0	0.0	0.1	0.0	0.1	0.5	0.1	0.2	0.0	0.0	0.0	0.0	0.0	0.0	0.0	0.0	0.0	0.0	0.0	0.0	0.0	0.0
AcMADS23	7.1	6.9	6.4	5.6	5.9	5.9	14.7	9.2	8.6	10.8	8.6	7.4	15.7	7.9	6.8	6.3	7.2	8.1	9.7	9.4	11.8	10.8	11.1	11.3	11.7	16.3	9.7
AcANR1c	0.0	0.2	0.0	0.0	0.1	0.2	0.4	0.1	0.1	0.6	0.2	1.0	0.5	0.2	0.1	0.1	0.3	0.0	0.4	0.1	0.5	0.7	1.3	1.4	2.0	4.6	4.9
AcFLC1	52.0	45.0	32.8	28.3	51.1	44.7	73.3	19.8	24.0	20.9	31.4	53.5	41.1	38.2	43.7	85.9	46.0	56.5	50.5	95.9	45.6	53.0	49.9	40.7	45.4	59.8	54.7
AcSEP3	36.6	45.0	92.0	67.6	115.2	135.0	710.5	83.6	73.4	68.6	90.7	149.7	180.6	190.0	135.0	193.8	124.6	237.6	198.4	217.3	156.0	146.3	147.0	137.5	149.1	204.6	128.4
AcAGL6	475.7	341.0	392.0	378.1	393.2	342.2	297.5	9.7	12.5	8.2	11.2	46.3	51.2	23.6	27.0	30.0	29.0	39.6	43.0	37.9	175.3	167.8	150.9	207.5	229.2	219.4	164.8
AcSOC1b	0.2	0.6	0.4	0.4	0.0	0.2	1.6	0.4	1.2	15.2	5.7	3.5	9.3	2.0	0.2	1.8	1.1	1.4	1.7	0.9	6.0	5.3	2.5	2.0	3.4	2.3	2.7
AcSOC1c	1.4	2.5	3.5	2.4	2.3	3.3	15.1	4.9	4.2	6.2	6.1	10.2	21.7	13.4	8.7	15.6	9.0	15.7	20.1	16.1	6.1	4.5	4.8	2.9	1.9	4.0	2.3
AcSOC1d	0.0	0.0	0.0	0.0	0.1	0.1	0.1	0.3	0.4	0.8	1.9	0.9	5.3	0.0	0.0	0.1	0.5	0.1	0.6	0.3	0.0	0.1	0.1	0.2	0.3	0.2	0.1
AcSEP1	85.6	59.8	138.5	124.0	40.3	28.8	162.7	7.3	5.5	4.8	7.0	11.8	12.7	41.5	41.6	64.4	35.4	67.6	43.5	66.3	8.0	8.9	6.6	6.8	8.3	11.7	6.1
AcAGL12b	1.8	2.3	3.8	3.3	0.2	1.9	8.8	5.5	1.7	0.2	0.5	0.7	0.7	0.3	0.4	2.0	0.5	0.4	0.8	3.7	0.2	0.3	0.1	0.2	0.1	0.1	0.2
AcMADS34	0.0	0.0	0.0	0.0	0.0	0.0	0.0	0.0	0.0	0.0	0.4	0.8	0.2	0.0	0.0	0.0	0.0	0.0	0.0	0.0	0.0	0.0	0.0	0.0	0.0	0.0	0.0
AcFLC2	0.2	0.3	0.3	0.1	0.5	0.3	0.2	2.7	1.7	1.1	2.8	0.6	7.7	0.3	0.2	0.2	0.5	0.5	0.7	2.1	0.4	0.7	0.6	0.9	0.8	1.4	1.6
AcPI	10.4	22.9	11.0	6.6	197.1	319.3	44.0	112.1	161.3	196.5	329.4	292.8	182.7	151.5	194.1	170.8	224.0	206.2	197.5	182.2	14.1	15.7	17.0	14.9	13.7	20.2	18.4
AcANR1d	0.0	0.0	0.0	0.0	0.0	0.0	0.0	0.0	0.0	0.0	0.2	0.0	0.0	0.0	0.0	0.0	0.0	0.1	0.1	0.0	0.0	0.1	0.0	0.0	0.0	0.1	0.0
AcMADS38	0.0	0.1	0.1	0.0	0.2	1.4	0.0	0.0	0.0	2.8	5.1	0.2	0.0	0.0	0.0	0.0	0.0	0.0	0.0	0.0	0.0	0.1	0.1	0.9	2.1	3.3	4.2
AcAP3	0.1	0.1	0.1	0.0	16.0	12.7	1.2	1.0	1.5	2.6	7.1	10.5	3.8	9.4	5.4	11.8	7.6	12.2	7.1	8.0	1.5	1.5	2.0	1.2	1.8	1.4	1.4
AcMADS40	10.9	16.6	12.9	12.0	12.6	14.1	16.4	15.6	16.1	20.2	19.1	13.8	8.4	10.7	11.7	9.6	14.1	9.1	8.4	7.7	11.3	11.9	15.3	12.6	15.8	10.0	15.5
AcSVP3	3.2	2.1	1.8	2.5	1.3	1.3	0.2	0.1	0.3	0.4	0.1	1.6	1.3	0.8	0.4	2.5	1.2	1.1	1.2	2.6	0.0	0.1	0.1	0.1	0.1	0.0	0.0
AcMADS42	0.1	0.1	0.0	0.0	0.1	0.1	0.0	0.0	0.0	0.0	0.1	0.0	0.0	0.0	0.0	0.0	0.0	0.2	0.0	0.1	0.0	0.1	0.1	0.1	0.0	0.1	0.1
AcSOC1e	0.0	0.0	0.0	0.0	0.3	0.2	0.0	0.8	1.4	2.5	4.3	3.8	15.3	0.0	0.2	0.5	2.1	0.8	2.5	1.3	0.2	0.1	0.1	0.8	0.5	0.8	0.6
AcANR1e	0.0	0.0	0.0	0.0	0.0	0.0	0.0	0.0	0.0	0.0	0.0	0.0	0.0	0.0	0.0	0.0	0.0	0.0	0.0	0.0	0.0	0.0	0.0	0.0	0.0	0.0	0.0

Table S4. List of TPM value of ABCDE model genes in four representative pineapple floral parts (sepal (S1-S3), petal , stamen, pistil , and ovule (S1-S3)) at different developmental stages

	Sepal_1	Sepal_2	Sepal_3	Petal	Stamen	Pistil	Ovule_1	Ovule_2	Ovule_3
AcAG	8.186211	12.076561	2.188171	1.297335	611.396606	286.068909	375.707733	233.428802	344.144257
AcAGL11a	1.416363	0.540011	0	0	0.950804	21.792984	805.119629	99.889046	114.208054
AcAGL11c	2.409096	0.402182	0.279012	0	14.997993	2.780759	1100.841675	451.746185	322.414276
AcFUL1	251.734436	173.947632	120.215485	15.662314	44.306515	58.44532	67.74942	84.912529	227.507721
AcFUL2	1.917681	0.500571	0.195296	13.225686	3.531693	8.983928	0.789622	0.165155	0.131564
AcAP3	3.112243	0.103706	0	186.941849	144.678467	108.90638	2.941939	0.293431	0.314709
AcBS	6.311272	0	0	0	10.021465	1.446927	334.144775	44.373417	0
AcPI	26.064533	15.140951	7.544228	365.454742	410.719208	427.315033	51.612328	42.9762	25.628199
AcAGL6	421.982666	315.277252	521.918335	398.259949	22.199963	9.666361	93.956963	91.479332	85.477829
AcSEP3	90.44912	109.871208	102.1101	196.484879	198.697067	224.710999	320.52475	650.797058	324.481049
AcSEP1	75.556793	102.724907	100.392685	49.037189	15.341432	53.247494	31.081751	36.270744	37.980759
AcAGL11b	0.316978	0	0	0	16.102308	111.411087	68.176971	17.927546	13.215206

Table S5.Primers used in this study

Gene ID	Primer Label	Sequnce 5'-3'
Aco015105.1	Aco1-A_F	CAAAGTAGCTGCCAAGAGTACA
Aco015105.1	Aco1-A_R	CTTGATGCTAAGTGTTCCCAAATC
Aco017589.1	Aco2-A_F	GGTTGTTCGTCACAGGAAGTA
Aco017589.1	Aco2-A_R	CATAGACTGGGTGCTCTTCTTT
Aco012428.1	Aco3-A_F	GTGAGGACTATGGGAAGCTAAAG
Aco012428.1	Aco3-A_R	GTTGTTGCTCCAGTTGTTGAAG
Aco009993.1	Aco4-B_F	TCGAAATCAAGAGGATCGAGAAC
Aco009993.1	Aco4-B_R	GCGGCTGGAGAAGACAATTA
Aco004839.1	Aco5-A_F	CCCTCATTATCTTCTCCACCAAG
Aco004839.1	Aco5-A_R	GCATAGGAGTAGCGCTCATAAC
Aco019365.1	Aco6-A_F	AGGAGCTGATCCCGATAGAA
Aco019365.1	Aco6-A_R	GTTCTCCTCCTCCAACAATCTC
Aco015487.1	Aco7-A_F	CCCACTCTGCAAATAGGGTATC
Aco015487.1	Aco7-A_R	ACCCAACCAAGCATGAAATTG
Aco017563.1	Aco8-A_F	CCACTGAGACACAGAGCAATTA
Aco017563.1	Aco8-A_R	TGGAATCAAATCCTCACCAAGA
